# A hybrid neural network for large-scale expressway network OD prediction based on toll data

**DOI:** 10.1371/journal.pone.0217241

**Published:** 2019-05-23

**Authors:** Xin Fu, Hao Yang, Chenxi Liu, Jianwei Wang, Yinhai Wang

**Affiliations:** 1 Department of Economic and Management, Chang’an University, Xi'an, Shaan Xi, China; 2 Department of Civil and Environmental Engineering, University of Washington, Seattle, WA, United States of America; Central South University, CHINA

## Abstract

Accurate Origin-Destination (OD) prediction is significant for effective traffic monitor, which can support operation decision in traffic planning and management field. The enclosed expressway network system like toll gates system in China can collect mounts of trip records which can be gathered for OD prediction. The paper develops a novel neural network, which is named Expressway OD Prediction Neural Network (EODPNN) for toll data-based prediction. The network consists of the following three modules: The Feature Extension Module, the Memory Module, and the Prediction Module. In the process, the attributes data which can reflect the city attribute such as GDP, population, and the number of vehicles are considered to embeded into the notwork to increase the accuracy of the model. For the applicability improvment of the model, we categorize the cities in multiple classes based on their economy and population scales in this paper, which can provide a higher accurate prediction of OD by EODPNN. The results shows that, comparing to the traditional model like ARIMA and SVM, or typical neural networks like Bidirectional Long Short-term Memory, the EODPNN delivers a better prediction performance. The method proposed in this paper has been fully verified and has a potential to transplant to the other OD data-based management systems for a more accurate and flexible prediction.

## Introduction

The origin-destination (OD) data is the fundamental source of transportation planning and management research no matter in urban or rural road systems [1]. Taking advantage of trip information based on OD data, many studies have significant achievements in traffic flow analysis and demand recognition [2]. In practice, OD prediction can be applied to multiple traffic flow and infrastructure management in expressway such as performance evaluation, investment decision-making, traffic volume balancing, deployment of personnel and resources and congestion mitigating [2,3,4]. However, the attributes of the OD data like continuity and granularity have a high impact on the final result, which indicates a reliable and concise method to collect the OD data is significant.

The generation of the OD data of expressway usually faces considerable difficulties [2], traditional methods like field survey and observation methods are not applicable to expressway condition. However, the enclosed expressway system in China provides (i.e., vehicles must pass through the toll gates when they enter or exit the expressway) a favorable condition to record the OD data. In the process, the toll data record generated by the toll gates contain detailed information about trips including vehicle type, entry and exit gates, entry and exit time, and toll fee. Therefore, the entry and exit toll gate sites can be considered as the origins and destinations of the trips separately within the network. As a result, the data generated by the toll system can be regarded as a kind of OD data essentially [5], and it can reflect the spatial characteristics of the traffic flow in the enclosed network.

Many studies have proved that the enclosed system generated OD data has satisfied performance in numeric applications of the expressway, which has been applied in many expressway related flow analytics. The OD data generated by trip records and vehicle information in toll gates can be used for traffic process identification, demand characteristics understanding [3], mobility performance evaluation [5], the traffic flow parameters estimation [6], travel time prediction and reliability evaluation [7,8] in the network. Additionally, some studies focus on the weight data in the toll system, which can assist the researchers to learn more about the vehicle types in the network [9].

In review of the existing studies, multiple attempts have been made for OD prediction in the enclosed expressway network, of which are based on Automatic Vehicle Identification (AVI) system [4] or loop data [1]. For the studies employ toll data (or vested OD data), we can classify them into three categories:

1. Linear theoretical models, based on historical average models, time series models and Kalman filtering models [4,10,11]
2. Nonlinear statistics models, based on nonparametric regression models and chaotic theoretical models [12,13]
3. Machine learning prediction models, based on neural network, support vector machine etc. [14,15,16,17,18].

In summary, existing studies, especially the neural network modeling method has made great progress in expressway OD prediction, however, further exploration is still needed on the following issues:

1. Most studies considered the case of one single road only [14,15] or a simple road network [17], while the model design research for large-scale, complex road networks is rare.
2. Some studies have carried out the design and application of neural network models based on link counts data [16], but the applicability of OD prediction at different time granularities still needs further discussion.
3. Some studies have realized neural network model design for OD prediction in the selected link instead of the complete network [18], however, to understand the characteristics of OD in practice, the network-scale study is necessary.
4. For OD prediction based on an artificial neural network, the influence of city attributes like the economy and population factor is rarely considered, which may impact the accuracy of the results neural network modeling.

The artificial neural networks have great advantages in dealing with related issues such as traffic flow/speed prediction[19] and traffic incident prediction [20,21]. The ANN like Recurrent Neural Network (RNN) was widely used in the transportation field for traffic flow estimation and prediction, some studies have also pointed out that some types of ANN have good advantages in large-scale network prediction while retaining the fine-scale structure[22]. RNN is a well-known kind of neural network can model sequential information by maintaining a chain-like structure and internal memory with loops [23] and is widely used in traffic information prediction. Taking advantage of RNN, a series of research on travel behavior recognition, trajectory prediction [24,25,26,27] have been developed recently. However, one technology challenge blocks the progress: the chain-like structure and deep loops deteriorate the process of model training, which lead the loss function (i.e., the deviance between the training results and the true records) of RNN hard to converge. There are multiple attempts for the difficulty and the Long Short-Term Memory (LSTM) neural network [28] is one of the successful attempts.

The LSTM neural network firstly proposed in 1997 is a special form of RNN that is capable of learning long-distance dependencies. Compared with the other neural networks, LSTM has better applicability in processing sequence data and identifying trends[29]. A multi-layered LSTM was introduced to present an end to end sequence learning that makes the minimal assumptions on the sequence structure [30]. Therefore, the translation result can be closer to the nature language with fewer misunderstandings. In the application of traffic data prediction field, the LSTM is also proven to handle spatio-temporal data. A Spatial-Temporal LSTM network was once used to capture the transportation trajectories features, which can memory and summarize the changes of GPS data sequence [31]. Another point to note is the LSTM applied in computer vision and machine translation filed shows that it can deal with several different data sequences and memory sequence characteristics[32].

In this paper, we put up with a hybrid network named Expressway OD Prediction Neural Network (EODPNN), and the remaining parts of this paper are organized as follows.: the second part is a description of the expressway toll data, the third part is the EODPNN structure, the fourth is the results and discussion and the fifth is the application and future implementation. The conclusion is located at the end of the paper.

Our contribution mainly located in four aspects:

1. A neural network called EODPNN is raised, which consists of three parts: Feature Extension Parts, Memory Module and Prediction Module. This model can realize high-precision prediction of large-scale expressway network (provincial level) based on toll data.
2. A large-scale, real provincial-level data set is trained and tested in this neural network and the performance is encouraging and promising. For every 15 minutes, the mean absolute error (MAE) is 2.46 vehicles and the mean absolute percentage error (MAPE) is 9.75%.
3. Four-time interval of OD prediction is tested, and the accuracy is steady and high. When the time interval is 15 minutes, the OD prediction accuracy is best with the MAE equaling to 2.46 vehicles.
4. Four different scales of cities: metropolis, big sity, medium city and small city are tested and the results show that the EODPNN has the ability to handle the express way OD prediction task located in different kinds of cities.

## Data description

### Toll data

The primary purpose of toll data collecting is to fully document the individual trip process and related details of the vehicle's use of the road to support charging behavior. The toll amount of a complete driving process usually needs to be determined by factors such as distance, vehicle type (sometimes including weight), driving time, etc. The typical data structure of a typical charging record (take Guangdong Province as an example) is shown in Table 1. Some fields not related to this paper are not listed here, such as axle weight, payment method, whether it is a free vehicle, data upload time, etc.

**Table 1:** Toll data structure.

Field Name	Field Type	Data Example	Remarks Example
Entry Gate No	Int	006	
Entry Lane No	Int	12	
Entry Time	Date	2017/9/1 20:54:00	
Exit Gate No	Int	002	
Exit Lane No	Int	2	
Exit Time	Date	2017/9/1 22:31:00	
Exit Lane Type	Int	1	‘0’-ETC, ‘1’-Non_ETC
Vehicle Type	Int	5	
Vehicle License	String	“A96534”	
Vehicle Kind	Int	0	‘0’-Car, ‘1’-Truck

The toll system will generate a large amount of flow data in a short period of time. Due to various situations, the toll data will generate abnormal data which may affect the result. Data for the following cases will be deleted in this paper to reduce interference.

- Data loss: Data loss: unable to reflect the OD information of a trip.
- Data redundancy: multiple records of data reflects a same trip.
- Data anomaly: Data records are contrary to normal trip rules, including entering and leaving the expressway from the same gate, the travel time is zero, etc.
- Data low precision: Data cannot be identified as a specific toll gate site, and the OD information cannot be extracted.

Although the traffic records in toll data are continuous in time, in order to observe the different prediction effects under typical time period separation periods, in this paper, we refer to some similar research practices[33], the toll data are combined use the same OD pair and summarized into 4 different time intervals: 15 minutes interval, 30 minutes interval, 45 minutes interval, and 60 minutes interval. In the model verification section, differences in model performance at varied time intervals will also be discussed.

### Attributes data

The interaction between traffic flow and the external environment has been recognized by many studies. Right attributes is significant to characterize the external environmental factors associated with traffic flow, which is helpful for flow pattern understanding[34]. In this paper, some traffic-related attributes are employed to collaborate with historical traffic OD data to explore its impacts. In a large spatial granularity, the characteristics of the nodes (cities) of the traffic network are usually related to three factors, economy situation, the total scale of potential travel (generation and attraction), and the achievability of travel[2][3][5][6]. To describe the three factors, the paper employs three indicators: GDP, population and the number of vehicles. The three attributes data can be obtained by matching the geographic coordinates of the toll gate with the city’s administrative boundary to clarify the attribute characteristics of each toll gate. As the neural network input, we summary these three attributes as a whole set, called CityID.

## Network structure of EODPNN

### Definition

#### Definition 1, OD Pair

**Fig 1:**
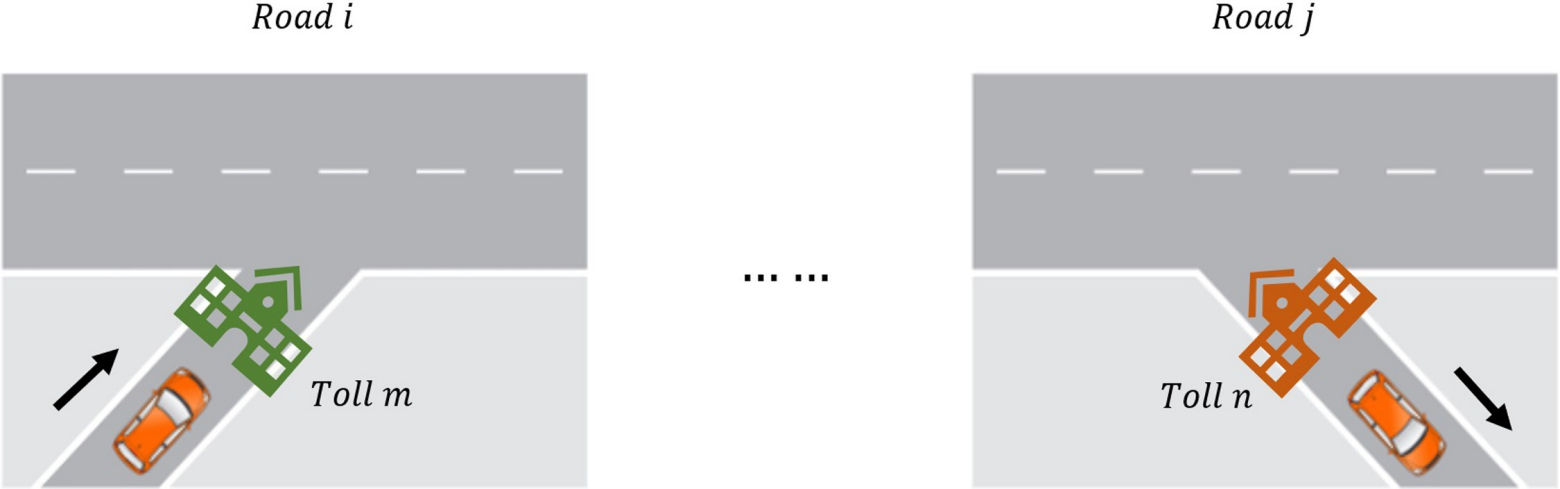
Expressway OD pair representation.

As shown in Fig 1, use $P_{im\_jn}^{\;t_{k}}$ to shows an OD pair generated by expressway toll gates at the time $t_{k}$, which means a vehicle is entering the freeway system from the road *i*, toll *m* and exit from the toll *n* on road *j*. Use ${NP}_{im\_jn}^{\;t_{k}}$ represent the total number of vehicles went through this OD for a certain time section *t*_*k*_.

#### Definition 2, generally attributes representation

In addition to the three macro attributes, the micro-performance of traffic flow is also affected by factors such as weather, periodic fluctuation and travel time selection etc., which can be considered as auxiliary variables to be able to make more detailed predictions[19][29][33]. To capture the more detailed flow variation pattern, we have included the above three factors as generally attributes and incorporated them into the prediction network model. The weather (*weatherID*), week date (*weekID*), time stamp (*timeID*) and city attributes *(cityID*, *including* GDP, population and the number of vehicles) are used as a set of general attributes. All these four attributes consists of an attributs set $A_{im\_jn}^{t_{k}}$, which belongs to a certain OD pair $P_{im\_jn}^{\;t_{k}}$ at the time slot *t*_*k*_. The attributes normalization and embedding strategies are discussed in the next part.

### Network establishment

**Fig 2:**
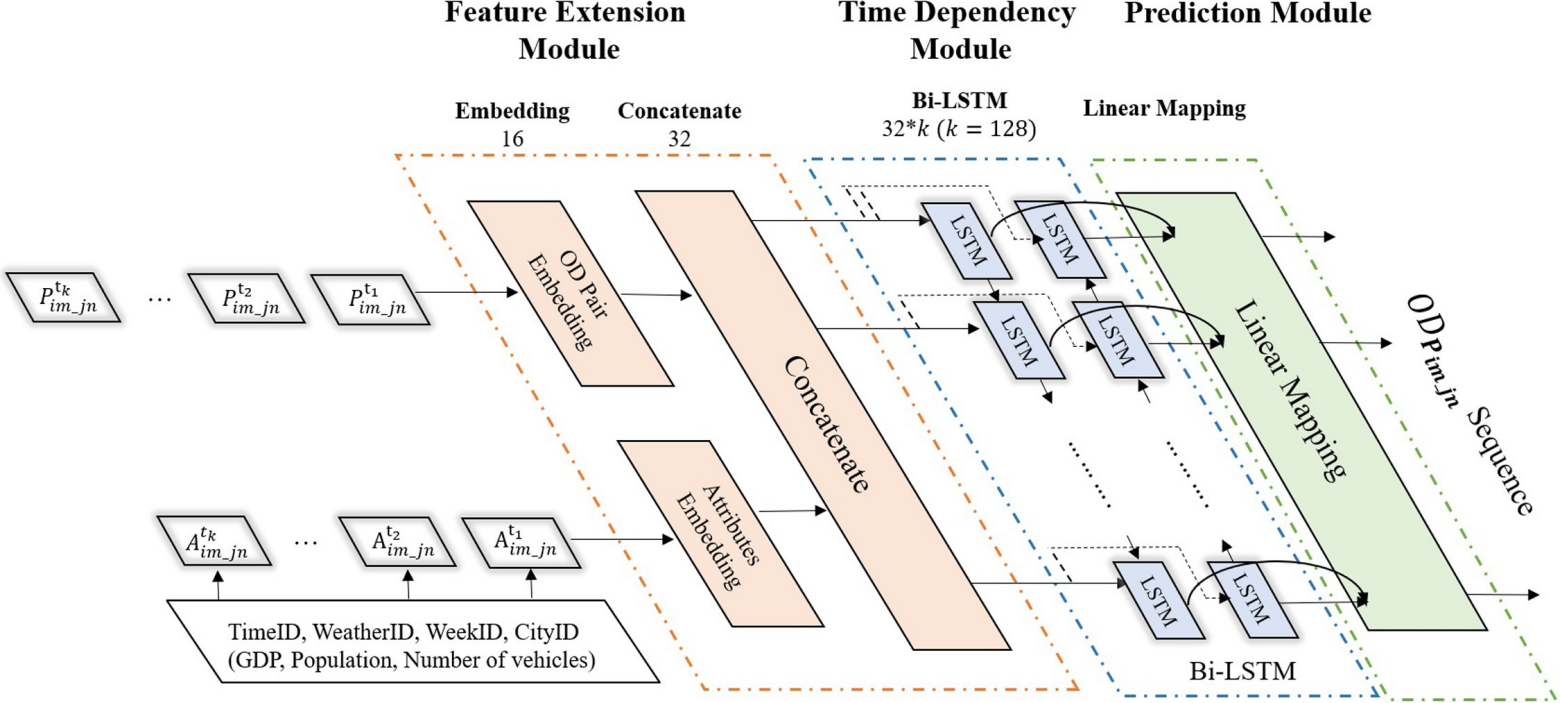
Express way OD prediction neural-network (EODPNN) architecture.

As is well-known, The OD data in a given time-period is usually presented in the form of a two-dimensional matrix, while the cell values represent the traffic flow between Origin and Destination [18]. The Neural-Network used for predicting this OD matrix is a Modular Plug-in Neural Network. The architecture shows in the Fig 2. Basically, it is consisted of three modules, the Feature Extension Module (FEM), the Memory Module (MM), and the Prediction Module (PM). The Feature Extension Module (FEM) is responsible for extract and convert features of a serious of OD pair, using embedding and concatenate method. Memory Module is based on Bi-LSTM to capture the time dependency of the OD pair changes. The Prediction Module is responsible for converting the neural network out put into OD flow sequence.

### Feature extension module (FEM)

#### Normalization & embedding& concatenate

As mentioned before, the OD distribution of express way is affected by tons of factors, such as time, location, weather situation, the located city economic situation, the located city population etc. An effective method to combine these and incorporate these factors in our neural network.

Here, GDP, Population, Number of vehicles, weather condition (rainy/snowy/sunny etc.), the time stamp (0–96) are necessarily added here. However, the format of these factors is not fit to neural network directly. For the GDP, Population, Number of vehicles used here, a normalization process is necessary. Here, researchers use the mean-standard method. The Mean-SD is used to avoid the shifting of the weights in the network caused by extreme values and the training process will be accelerated either.

$${\hat{x}}^{\left(k\right)}=\frac{x^{\left(k\right)}-E\left[x^{\left(k\right)}\right]}{\sqrt[{2}]{Var\left[x^{\left(k\right)}\right]}}$$ (1)

Embedding is an effective way to transform discrete categorical attributes into a low-dimensional vector. Here, to achieve the process, we use a mathematical mapping method form each categorical into a vector of $R^{m*1}$in this paper. In our research, $P_{im\_jn}$ is embedding into a space of **R**^16^, called $V_{P_{im\_jn}}$; and the attributes sets are also embedding into a space of **R**^16^, called $V_{{attribute}_{im\_jn}}$.

Concatenate here is used to combine both information vector into a whole vector before capture the time dependency. ⨀ represents concatenate:

$${\hat{P}}_{im\_jn}=V_{P_{im\_jn}}\;⊙V_{attribute_{im\_jn}}$$ (2)

### Memory module (MM)

In many real-world tasks, especially traffic flow prediction, in OD prediction problems, the input of the network is not only related to the input at the current moment, but also related to the output of the past period of time. Such prediction problems often have the length of time series data is generally not fixed, the correlation between adjacent data is very strong, and there is an accumulation effect. Recurrent Neural Network (RNN) is a kind of neural network with short-term memory ability. In a circulating neural network, neurons can not only receive information from other neurons, but also accept their own information to form a network structure with loops.

**Fig 3:**
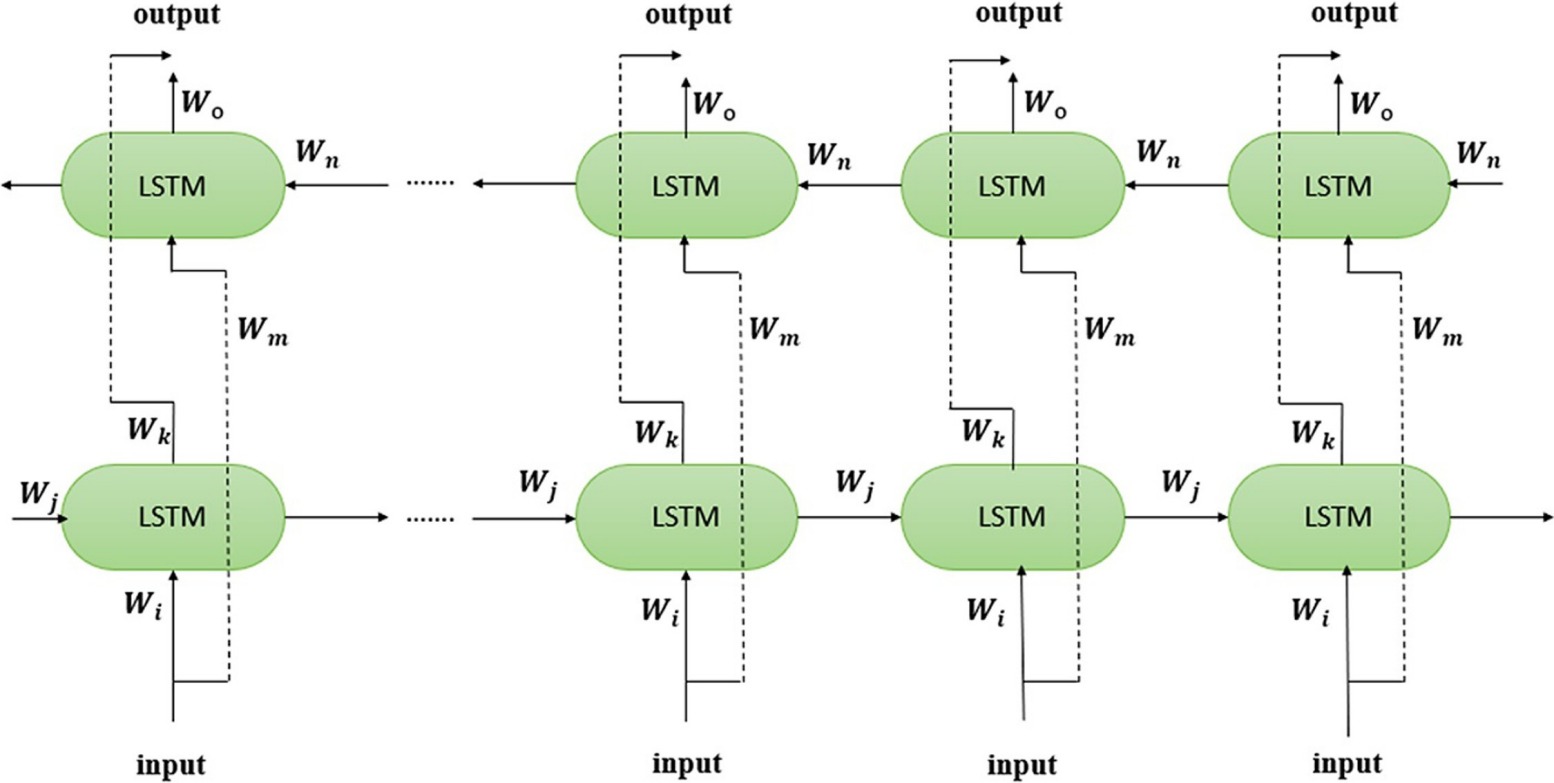
Bidirectional LSTM (Bi-LSTM) network.

Bidirectional LSTM (Bi-LSTM) is a novel improved version of LSTM network used in EODPNN. In Bi-LSTM, two LSTM layers pass the data sequence on two opposite directions, one forward direction and another backward direction. The output has concatenated both layers into a long vector including both direction time dependency features. Both former step input and later step input information are combined. The Fig 3 below shows Bi-LSTM structure. Six unique weights are used repeatedly here, which are correspond to: input to the forward and backward hidden layers (*w*_*i*_, *w*_*m*_), and hidden layers to the hidden layer itself (*w*_*j*_,*w*_*n*_), forward and backward hidden layer to output layer (*w*_*k*_, *w*_*o*_).

In our module, the output sequence *Output*_*BiLSTM*_ can be shown as

$$Output\_FP_{BiLSTM}=\sigma_{rnn}\left(\;W_{i}\cdot Input_{FP}+\;W_{j}\cdot h_{i-1}+\;W_{k}\cdot Output_{FP}\right)$$ (3)

$$Output\_BP_{BiLSTM}=\sigma_{rnn}\left(\;W_{m}\cdot Input_{BP}+\;W_{n}\cdot h_{i-1}+\;W_{o}\cdot Output_{BP}\right)$$ (4)

$$Output_{BiLSTM}=\;Output\_FP_{BiLSTM}⊙Output\_BP_{BiLSTM}$$ (5)

**Fig 4:**
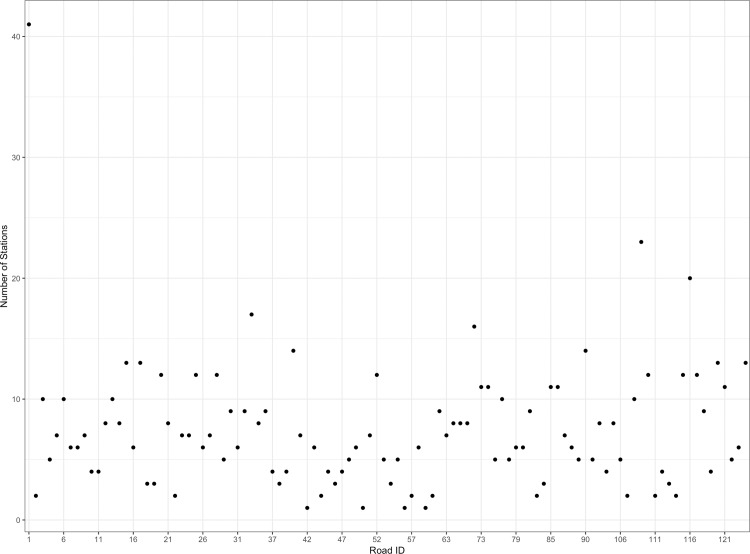
Summary of toll gates distribution.

$h_{i-1}$is the hidden state represent after processed the *i-*1_th time stamp in BiLSTM network. $\sigma_{rnn}$ is the activation function researcher used here.*W*_*i*_, *W*_*j*_, Wk,Wm, *W*_*n*_, *W*_*o*_, are learnable parameter matrices located in two different directions and they are all illustrated in the Fig 4.

### Prediction module

This module is responsibility for transforming the vector into a $N_{P_{im\_jn}}$ data sequence. Four Linear Mapping Layer are used here, transform the vector from 32 to the output sequence. The active function of each layer if is Leaky ReLU function.

### Model training

Here the mean absolute error (MAE), the mean absolute percentage error (MAPE) and root mean squared error (RMSE) are used to train this network based on different training strategy. MAE measures the average magnitude of the errors of OD prediction numbers. RMSE is a quadratic scoring method which also used to measure the average magnitude of the OD prediction distribution and useful to represent the large errors and the extreme values. MAPE represents the percentage of the error and always use as a measurement to show the overall performance of the whole network. *ε* is used to prevent the 0-denominator and here *ε* is 3. Three loss-function in details:

$$MAE=\frac{1}{N}\sum_{i,m,j,n}\left|N_{P_{im\_jn}}\;-\hat{N_{P_{im\_jn}}\;}\right|$$ (6)

$$MAPE=\;\frac{1}{N}\sum_{i,m,j,n}\left|\frac{N_{P_{im\_jn}}\;-\hat{N_{P_{im\_jn}}\;}}{N_{P_{im\_jn}}\;+\varepsilon }\right|*100\%$$ (7)

$$RMSE=\;\sqrt[{2}]{\frac{1}{N}\sum_{i,m,j,n}{\left(N_{P_{im\_jn}}\;-\hat{N_{P_{im\_jn}}\;}\right)}^{2}}$$ (8)

## Experiment

### Research area description

In this paper, we use the expressway network toll data of Guangdong province for empirical study. This data set is collected and managed by the Guangdong Provincial Network Charge Settlement Center and provided by the Guangdong Provincial Road Transportation Administration. Guangdong Province is a coastal province located in southeastern China. It has 21 prefecture-level cities and 119 county-level administrative districts. The expressway network in Guangdong Province is one of the most important transportation network subsystems in South China. The entire road network’s main body is composed by nine vertical roads, five horizontal roads and two rings roads, and the total mileage of network in 2017 is 7673 kilometers. According to the geographical location, the entire network is divided into four sub-areas of the Pearl River Delta region, the eastern region, the western region and the northern region. Among them, the expressway density in the Pearl River Delta region is at a very high level, second only to New York, USA. It is the first in Asia and the second in the world.

According to the different construction and management subjects, the entire expressway network of Guangdong is divided into 115 road sections for charging, with a total of 953 toll gates. Since some toll gates are virtual sites, or separate bridges or tunnel toll gates, and no actual vehicles use these gates to enter or leave the expressway, the actual amount of toll road sections used in this paper are 104, and the total number of toll gates are 781. The distribution of the toll gates in each section is shown in Fig 4. The data used in the paper covers 30 days (April 2017) with average 3.8 million toll records every day. Note that we delete the abnormal data according to the cases mentioned in the data description section when processing the network training and model validation.

**Fig 5:**
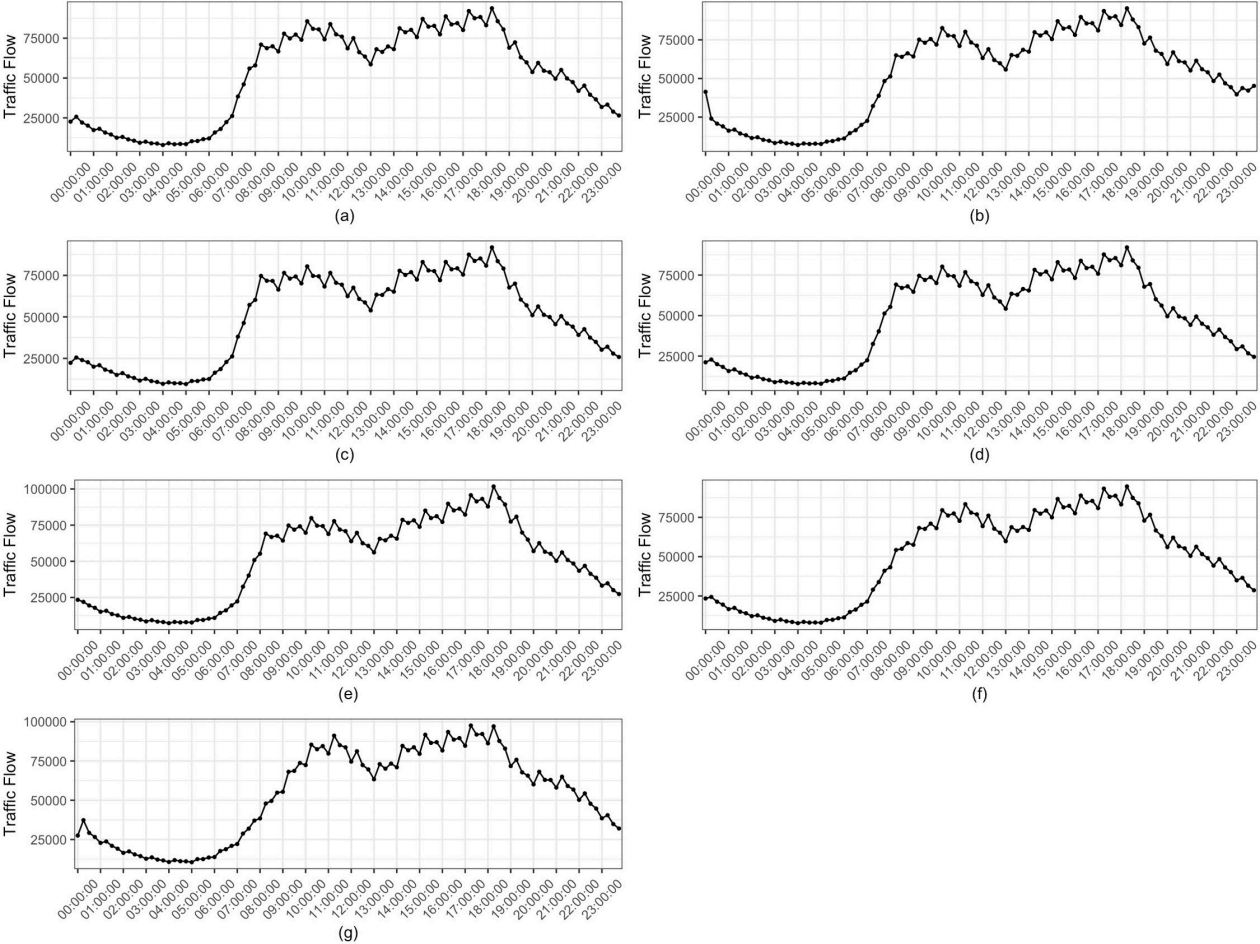
Daily average flow pattern. (a) Monday. (b) Tuesday. (c) Wednesday. (d) Thursday. (e) Friday. (f) Saturday. (g) Sunday.

**Fig 6:**
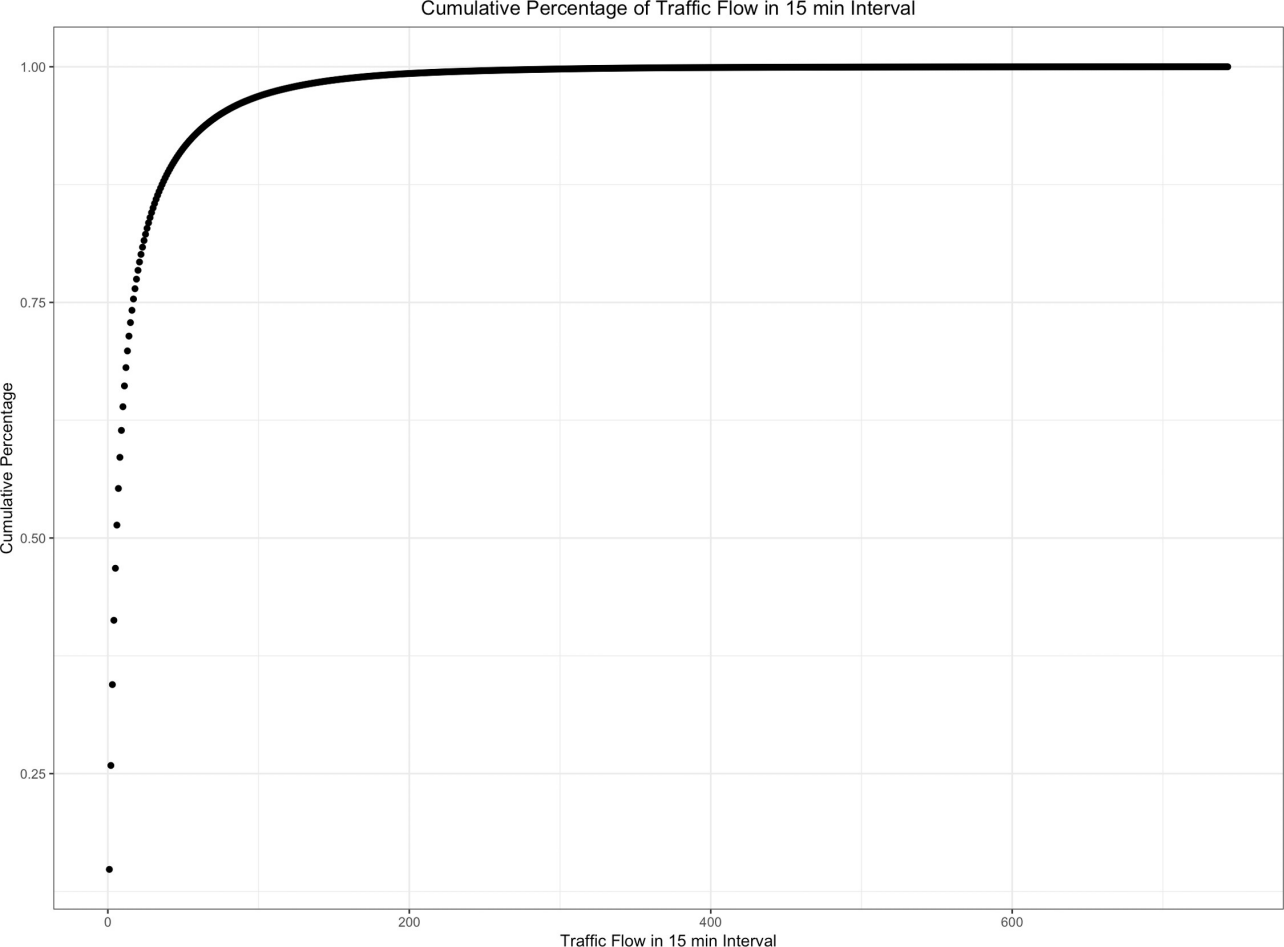
Cumulative flow distribution of OD pairs in network.

As to the distribution of OD pairs, Fig 5 is a plot of the network’s daily average flow pattern over time for a week in the entire network. And Fig 6 is the plot of the cumulative flow distribution of OD pairs based on the 15 mins interval. The results show that the overall traffic distribution of this network has a typical time-varying pattern in one day or one week.

### Environment description

EODPNN is built with PyTorch 3.1, a well-known AI platform. The training computer equipped with a NVIDIA 1080ti and one Intel Core i7 CPU. The operation system is Linux Ubuntu 17.04.

### Performance evaluation

The results of model training process looks reasonable, and the network performance shows in the following Table 2:

**Table 2:** Network performance comparison.

	MAE (veh/15min)	RMSE (veh/15min)	MAPE (%)
AVG	13.4	14.2	104.25%
ARIMA	17.96	19.39	57.9%
**SVM**	14.69	18.19	50.12%
RNN	9.13	9.44	33.48%
1 Layer-LSTM	7.08	7.85	27.74%
2 Layer-LSTM	4.12	5.16	16.25%
Bi-LSTM	3.67	4.08	14.71%
EODPNN	2.46	3.01	9.75%

• **AVG**: Calculate the average flow of each OD pair within a specific time interval simlply and use the mean as the predicated value.

• **ARIMA**: Employ ARIMA model to predict the flow of OD pairs within a specific time interval.

• **SVM**: Employ (SVM) model to predict the flow of OD pairs within a specific time interval.

• **RNN**: Take advantage of (RNN) to predict the flow of OD pairs within a specific time interval.

• **1 Layer LSTM and 2 Layer-LSTM**: Use one and two layers Long Short-Term Memory (LSTM) networks to predict the OD sequences. The result shows that a 2-layers LSTM is much better than one-layer [35].

• **Bi-LSTM**: Use bidirectional LSTM (Bi-LSTM) network without the Feature Extraction Module [36].

From the result, it’s clear to see that the LSTM network is EODPNN effective to do the OD prediction. And with the help of feature extension module, the MAPE accuracy is added 4.96% than only use the Bi-LSTM.

As mentioned in the introduction, people do OD prediction based on different time interval. EPONN is also trained by four different time interval data and evaluated separately. Here, researcher test data set based on the each trained EODPNN and the results show Table 3. The 15 minutes prediction has a highest accuracy about 9.75% overall MAPE, and 2.46 vehicles MAE, which is encouraging and promising. Also, EODPNN also has a steady and good performance in other different time intervals.

**Table 3:** Prediction performance for different time intervals.

Time interval	MAE	RMSE	MAPE
15 minutes	2.46	3.01	9.75%
30 minutes	4.02	5.04	12.32%
45 minutes	5.25	7.52	11.01%
60 minutes	8.91	11.89	11.24%

In normal conditions, the uneven development of cities in the same region leads to a large deviation in the prediction of OD data. In order to measure the performance of EODPNN in different situations, we divided the data into four different categories of cities: Metropolis, Big city, Medium City and Small City. Here, rules are according to the total amount of GDP, the total population and the number of vehicles owned by each city (Use the data of 2015).

Traditionally, metropolis likes Shenzhen and Guangzhou, with the top GDP volume in China and both more than 10 million permanent residents, also with the very high vehicles numbers. The second part is big city, includes: Foshan, Zhanjiang, Dongwan, with more than 6 million to 10 million people and relative high vehicle owners. The third part is medium city, such as Meizhou, Qingyuan etc. These cities hold a population of 3 million to 6 million and with a medium number of car owners. The last part is small city with less than 3 million people and relative a smaller number of vehicles. The comparison performance is showed in the Table 4.

**Table 4:** Prediction performance for different scale of city toll gates.

City	Examples	Evaluation index	Value (per 15min)
Metropolis	Gudang dong Shenzhen	MAE (Veh)	3.42
		RMSE (veh)	8.06
		MAPE (%)	14.67%
Big city	Foshan Zhanjiang Dongwan	MAE (veh)	2.26
		RMSE (veh)	2.42
		MAPE (%)	8.89%
Medium City	Meizhou Qingyuan etc.	MAE (veh)	2.14
		RMSE (veh)	2.73
		MAPE (%)	12.74%
Small City	Zhuhai Chaozhou etc.	MAE (veh)	2.81
		RMSE (veh)	3.42
		MAPE (%)	10.15%

From the results, metropolis prediction accuracy is relatively low, since parts of these two cities tolls with an extremely large number and huge vibration hard to predict. And also, tolls belong to a same city is very different. However, EODPNN has the ability to give a very high accuracy (with a under 3 vehicles MAE) prediction results except this two Metropolis.

## Application and implementation

In this study, we used the enclosed expressway toll data and corresponding attributes data to construct the EODPNN for implementing OD prediction with varied time granularity. From the perspective of application and implementation of the method, such prediction work will help to improve the operation level of the entire road network. In general, the toll amount of China's expressway usage is determined by the vehicle type (or weight) and mileage, which also indicates that there is a close relationship between the traffic volume within toll gates and the intensity and financial income, so, the OD prediction results with high accuracy and variable time granularity will have irrefutable impacts on the expressway network’s sustainability of finance and maintenance, and have a positive impact on operational and management improvements. The method proposed in this paper can predict the OD flow data of the whole expressway network based on toll data within a certain precision and accuracy range, therefore, it will be more practical and effective than some methods and experimental analytics which may provide prediction results for single certain toll gate or a road segment. In most cases, the toll system of expressway network is based on provinces. In a province's network, a relatively complete collection system for accumulating toll data has been formed, therefore, the method proposed in this paper can be transplanted into the toll data management system to provide multi-purpose predictions. It will help to develop financial policies for the entire network, including financing and maintenance inputs, and allocate resources within the network with greater efficiency. It can also achieve efficient segment traffic prediction based on OD prediction results, thereby reducing the cost of road segment flow observation and prediction. Other than that, we believe that another noteworthy issue of this work is to provide a large-scale network prediction solution based on distributed data. Although the data set formed by exclosed road networks is relatively rare, with the continuous improvement of survey and detection methods, the distribution data formed by vehicle trajectory data observation or large-scale travel surveys can be continuously obtained, and this method is also applicable for those scenarios. It will also facilitate the establishment of prediction networks based on other types of massive real-time data set and attributes data.

## Conclusion

In this paper, we put forward an expressway origin and destination (OD) prediction neural network (EODPNN) based on Bi-LSTM. City attributes and many general features are combined to improve the prediction accuracy. After training and testing EODPNN by using a provincial scale dataset, the results show the EODPNN with a steady and encouraging accuracy and the NN has the ability to predict OD based on various time intervals (15mins, 30 mins, 45 mins and 1 hour) with relatively high accuracy. Also, EODPNN has a bright future of implementation and artificial intelligent technology shows the power in transportation area again. Researchers will carry on improving the accuracy of EODPNN and further do more researches on expressway flow prediction using other advanced neural network.

## Supporting information

S1 File Attributes sample. This file contains the attributes data sample used in model training section presented in the paper.
(TXT)

S2 File Data field description. This file contains the field description of Attributes sample and OD Data sample.
(TXT)

S3 File OD Data sample. This file contains the OD Data sample used in model training section presented in the paper.
(TXT)

## References

[1] Mussone L, Grant-Muller S, Chen H. A Neural Network Approach for Motorway OD Matrix Estimation from Loop Counts. Journal of Transportation Systems Engineering and Information Technology. 2010;10(1):88–98.

[2] Miska M, Warita H, Kuwahara M. Analysis of Tokyo Metropolitan Expressway’s demand using ETC-OD data. Proceedings of Infrastructure Planning in Japan Vol. 2009; 39:1–4.

[3] Wang Y, Yang L, Geng Y, Zheng M. OD matrix estimation for urban expressway. Journal of Transportation Systems Engineering and Information Technology. 2010;10(2):83–87.

[4] Asakura Y, Hato E, Kashiwadani M. Origin-destination matrices estimation model using automatic vehicle identification data and its application to the Han-Shin expressway network. Transportation. 2000;27(4):419–438.

[5] Qu T, Li D, Wikander J. Evaluation of Mobility Performance with Toll Data for Jingshen Expressway in China. Transportation Research Record: Journal of the Transportation Research Board. 2014; (2451):77–87.

[6] Zhao N, Qi T, Yu L, Zhang J, Jiang P. A Practical Method for Estimating Traffic Flow Characteristic Parameters of Tolled Expressway Using Toll Data. Procedia-Social and Behavioral Sciences. 2014; 138:632–640.

[7] El Faouzi NE, Billot R, Bouzebda S. Motorway travel time prediction based on toll data and weather effect integration. IET intelligent transport systems. 2010; 4(4):338–345.

[8] Yamazaki H, Uno N, Kurauchi F. The effect of a new intercity expressway based on travel time reliability using electronic toll collection data. IET Intelligent Transport Systems. 2012; 6(3):306–317.

[9] Zhang H, Ni F. Study of Expressway Axle Load Spectrum Based on Toll Data of Jinghu Expressway. Journal of Testing and Evaluation. 2012 11 26;40(7):1220–1227.

[10] Wang H, Xiang Q, Lu J, Gao C, Liang C. Method of expressway ramp OD matrix calculation. Journal of highway and transportation research and development. 2005:108–111.

[11] Chang Y, Peng G, Yang X. Freeway OD matrices estimation with on/off ramps traffic counts [J]. Journal of Traffic and Transportation Engineering. 2003; 4:89–94.

[12] Du W, Zhao D, Liu Y. Calculation of the Expressway OD Matrix Based on Grey Markov Chain Model. International Conference on Transportation Engineering 2009; 2707–2712.

[13] Wang J, Wei G, Yang F, He Q. Expressway OD Matrix Estimation Based on the Fuzzy Optimization Grey Prediction Model. Intelligent Systems and Applications (ISA), 2nd International Workshop on 2010; 1–5.

[14] Wang K, Li J, Zhang M. Method of Expressway Ramp OD Matrix Estimation Based in Neural Network. Traffic and Computer. 2007;25(4):35–37.

[15] Liang C, Dong J, Cheng J. Calculation method of freeway OD matrix and statistics based on ramp detail. Journal of University of Shanghai for Science & Technology. 2010;32(5):471–474.

[16] Lorenzo M, Matteo M. OD matrices network estimation from link counts by neural networks. Journal of Transportation Systems Engineering and Information Technology. 2013; 13(4):84–92.

[17] Kikuchi S, Tanaka M. Estimating an origin-destination table under repeated counts of in-out volumes at highway ramps: use of artificial neural networks. Transportation Research Record: Journal of the Transportation Research Board. 2000; (1739):59–66.

[18] Remya KP, Mathew S. OD matrix estimation from link counts using artificial neural network. International Conference on Innovations in Civil Engineering. 2013;287–290.

[19] Ma X, Dai Z, He Z, Ma J, Wang Y, Wang Y. Learning traffic as images: a deep convolutional neural network for large-scale transportation network speed prediction. Sensors. 2017;17(4):818. 10.3390/s17040818 PMC5422179 28394270

[20] Tang J, Liang J, Han C, Li Z, Huang H. Crash injury severity analysis using a two-layer Stacking framework. Accident Analysis & Prevention. 2019 1 1; 122:226–238. 30390518 10.1016/j.aap.2018.10.016

[21] Zou Y, Zhong X, Tang J, Ye X, Wu L, Ijaz M, et al A Copula-Based Approach for Accommodating the Underreporting Effect in Wildlife‒Vehicle Crash Analysis. Sustainability. 2019 1;11(2):418.

[22] Yu H, Wu Z, Wang S, Wang Y, Ma X. Spatiotemporal recurrent convolutional networks for traffic prediction in transportation networks. Sensors. 2017 7;17(7):1501. 10.3390/s17071501 PMC5539509 28672867

[23] Jozefowicz R, Zaremba W, Sutskever I. An empirical exploration of recurrent network architectures. International Conference on Machine Learning 2015;2342–2350.

[24] Van Lint JW, Hoogendoorn SP, van Zuylen HJ. Accurate freeway travel time prediction with state-space neural networks under missing data. Transportation Research Part C: Emerging Technologies. 2005; 13(5–6):347–369.

[25] Elhenawy M, Chen H, Rakha HA. Dynamic travel time prediction using data clustering and genetic programming. Transportation Research Part C: Emerging Technologies. 2014; 42:82–98.

[26] Ye Q, Szeto WY, Wong SC. Short-term traffic speed forecasting based on data recorded at irregular intervals. IEEE Transactions on Intelligent Transportation Systems. 2012; 13(4):1727–1737.

[27] Karlaftis MG, Vlahogianni EI. Statistical methods versus neural networks in transportation research: Differences, similarities and some insights. Transportation Research Part C: Emerging Technologies. 2011; 19(3):387–399.

[28] Hochreiter S, Schmidhuber J. Long short-term memory. Neural computation. 1997; 9(8):1735–1780. 9377276 10.1162/neco.1997.9.8.1735

[29] Ma X, Tao Z, Wang Y, Yu H, Wang Y. Long short-term memory neural network for traffic speed prediction using remote microwave sensor data. Transportation Research Part C: Emerging Technologies. 2015 5 1; 54:187–197.

[30] Sutskever I, Vinyals O, Le QV. Sequence to sequence learning with neural networks. Advances in neural information processing systems.2014; 3104–3112.

[31] Zheng Y, Zhang L, Xie X, Ma WY. Mining interesting locations and travel sequences from GPS trajectories. Proceedings of the 18th international conference on World wide web 2009; 791–800.

[32] Vinyals O, Toshev A, Bengio S, Erhan D. Show and tell: A neural image caption generator. Proceedings of the IEEE conference on computer vision and pattern recognition 2015; 3156–3164.

[33] Lv Y, Duan Y, Kang W, Li Z, Wang FY. Traffic flow prediction with big data: a deep learning approach. IEEE Transactions on Intelligent Transportation Systems. 2015 4;16(2):865–873.

[34] Ma X, Zhang J, Ding C, Wang Y. A geographically and temporally weighted regression model to explore the spatiotemporal influence of built environment on transit ridership. Computers, Environment and Urban Systems. 2018 7 1; 70:113–124.

[35] Cui Z, Ke R, Wang Y. Deep Stacked Bidirectional and Unidirectional LSTM Recurrent Neural Network for Network-wide Traffic Speed Prediction. 6th International Workshop on Urban Computing 2017.

[36] Cui Z, Ke R, Wang Y. Deep Bidirectional and Unidirectional LSTM Recurrent Neural Network for Network-wide Traffic Speed Prediction. arXiv preprint arXiv:1801.02143. 2018.

